# *Strategic Team Science*: Scaffolded training for research self-efficacy, interdisciplinarity, diversity, equity, and inclusive excellence in biomedical research

**DOI:** 10.1017/cts.2021.810

**Published:** 2021-07-22

**Authors:** Yulia A. Levites Strekalova, Yufan Qin, Wayne T. McCormack

**Affiliations:** 1 Department of Health Services Research, Management & Policy, College of Public Health and Health Professions, University of Florida, Gainesville, FL, USA; 2 Clinical and Translational Science Institute, University of Florida, Gainesville, FL, USA; 3 Department of Mass Communication, College of Journalism and Communications, University of Florida, Gainesville, FL, USA; 4 Department of Pathology, Immunology & Laboratory Medicine, College of Medicine, University of Florida, Gainesville, FL, USA

**Keywords:** diversity, equity, inclusion, team science, workforce development, cross-disciplinary collaboration

## Abstract

Research collaboration is an essential research skill that promotes diversity and inclusion in research and requires comprehensive curriculum and instructional methods to provide early-stage trainees with low-risk, scaffolded experiences of collaborative research practice. *Strategic Team Science* is an instructional method that introduces biomedical science trainees to an inclusive way of thinking, capitalizes on the diversity of individual capabilities, and provides scaffolded experience of cross-disciplinary collaboration. Pilot results show that guided dialogues around *Strategic Team Science* increase research self-efficacy and interdisciplinary research orientation. Scaffolded collaboration dialogues allow students from diverse disciplines to engage actively and share ideas equitably.

## Rationale for Novel Instructional Method

Collaboration in science has grown rapidly and broadened the boundaries of research [1]. Its importance becomes more evident particularly in clinical and translational science efforts, in which research is more likely to be conducted in team settings involving multiple scientific disciplines [2]. The purpose of collaboration is to construct a shared view of the problem and possible solutions. Collaborative research requires not only the exchange of ideas, skills, and expertise but also diversity and equity in expressing ideas and world views, a high degree of joint attention, communication, interaction, mutual engagement, and co-elaboration of knowledge [1, 3]. Effective collaborative communication requires taking diverse perspectives and participating in the process of social knowledge production [3]. Consequently, the experience of collaborative research exposes team members to the diversity of perspectives and requires equity in voice in sharing ideas and clarifying differences [3]. These communication behaviors involve the development of mutual trust, effective conflict resolution, and psychological safety within the group [4–6]. Effective practice of research can be enhanced and supported by the focused development of skills needed to conduct effective collaborative research, such as social skills to build good relationships with team members, communication skills to facilitate exchange of knowledge and information, and cognitive skills to understand the issues coming from the interdisciplinary research questions [7].

## Unmet Educational Need

Research collaboration is an essential research skill that promotes diversity and inclusion in research and requires comprehensive curriculum and instructional methods to provide early-stage trainees with low-risk, scaffolded experiences of collaborative research practice. Collaborative research requires both understanding the boundaries of one’s own academic discipline and the ability to identify the points of connection with other disciplines. Yet, early-stage trainees often have evolving disciplinary identities and lack the depth of knowledge to identify complementary ideas and technologies [8]. Therefore, instructional strategies for collaborative research skill development need to balance supporting the knowledge that trainees bring from their disciplines and scaffolding for the interdisciplinary communication that promotes the diversity of opinions and equity of voice. Learning spaces where trainees can practice collaboration skills stand to enhance the curriculum and empower trainees to assume responsibility for their own learning [9]. Experiential learning theory views knowledge as a transformative and continuous experience and leads to the development of stable cognitive frameworks [10]. To be effective, learning experiences require concrete experiences of learned practices, reflective observations and abstract conceptualization about the experiences, and low-risk active experimentation which tests new application of learned practices [10, 11]. Experiential learning and guided practice of collaboration skills also provide the scaffolding for the development of translational research self-efficacy, a confidence to undertake and persist with clinical research tasks such as protocol development and publication [12]. Furthermore, confidence about individual research tasks also predicts trainees’ continuous involvement in research [12]. In the context of team science training, educational experiences for collaborative research practices need to draw attention to individual resources and existing research knowledge of each team member, while engaging teams in integrative and reflective dialogues to develop shared research objectives and projects. Building on the existing evidence for the effectiveness of integrative research dialogues [13] and strategic project planning [14], this brief report describes *Strategic Team Science*, an instructional method that introduces PhD trainees to an inclusive way of thinking to capitalize on the diversity of individual capabilities and to gain an experience of collaborating with strangers outside of their discipline.

## Target Audience

We have developed a *Strategic Team Science* workshop and implemented it as a 1-hour classroom session during a graduate-level team science course. Students were from multiple disciplines that could be broadly grouped as social behavioral sciences (e.g., communication, public relations, and health education) and biomedical sciences (e.g., biomedical engineering, physiology, and cancer biology). Subsequently, we made minimal revisions to the *Strategic Team Science* activity reported here and used it successfully in a team science workshop for junior faculty.

## Description of the Educational Method or Curricular Program

The framework for the *Strategic Team Science* workshop came from a rigorously developed and empirically validated method for strategically developing and implementing projects within loosely connected networks [14]. In line with the original approach, the *Strategic Team Science* session was framed by a forward-oriented appreciative question that asked the teams to consider: What would a successful collaborative project where multiple disciplines are represented look like? Students were divided into four teams of six to seven students. Teams were given cards with possible topics that represent broad wicked problems that are currently faced by the research community, *e.g.*, climate change, biodiversity loss, persisting poverty, the advancing obesity epidemic, and food insecurity. Those topics were kept intentionally broad so that the students could explore diverse collaboration ideas. The teams were instructed to briefly deliberate and choose one topic but not to start proposing any possible projects. After teams were formed and topics announced, we made a brief (7-minute) presentation that provided general information about strategic collaborations and resource networks. We also talked about the importance of psychological safety and equity of voice.

Next, the students received brief instruction for three steps to practice collaborative dialogues. *Step 1*: Students were instructed to talk first about their skills, knowledge, and access to resources that they are free and willing to bring for collaboration. In sharing their resources, we asked them to practice two considerations: (1) share one resource at a time, let the next person talk, and go through as many rounds of sharing as necessary to practice the equity of voice; (2) share only the resources they have free access to and are willing to share with anyone to open the opportunity for future collaboration queries. This step took about 15 minutes. *Step 2*: Students were asked to review the list of resources that their team had generated, pick one of the resources, and add complementary resources from every member of the team. This activity allowed the trainees to practice thinking innovatively while evaluating the resources and ensuring that every member of team is represented on the project in a meaningful way. For this step, students were asked to identify 3–4 different possible combinations of resources for future collaborative projects. *Step 3*: After about 15 minutes and once resource combinations and possible future projects started to emerge, students were instructed to vote on the projects based on two criteria: potential future impact should this idea be implemented, and the technical ease of starting to implement the projects. The goal of this voting activity was to help students identify the “Big Easy” project and to engage in continuous discussions to share the differences in opinions and understanding of the scope and complexity of emerging projects. Throughout the discussions, students were instructed to take notes of the shared resources, resource combinations, emerging projects, and the voting outcome to ensure that every voice was heard and the team created a tangible record of the dialogue. In total, teams took 35–40 minutes to move through the three steps of the Strategic Team Science dialogue, discussing ideas, identifying opportunities, and voting.

## Methods of Evaluation

We employed a pre- and post-session survey to evaluate the *Strategic Team Science* session. We hypothesized that scaffolded guidance through collaborative dialogues with those from different disciplines will have a positive effect on PhD trainees’ research self-efficacy (i.e., improve perceptions of their own research skills). We also hypothesized that *Strategic Team Science* dialogue will increase students’ orientation toward cross-disciplinary research (i.e., perceptions toward the importance of topics from other disciplines and interest in exploring those topics). Research self-efficacy was measured using a 12-item version of the Clinical Research Appraisal Inventory (CRAI-12) [12]. Although developed for clinical research, the scale assesses self-confidence of trainees in performing different aspects of research and focuses on the domains broadly applicable to science competencies and training outcomes, including data collection and analysis; reporting, interpreting, and presenting results; conceptualizing studies and collaborating; planning; funding; and protecting human subjects [12]. We also used the Research Orientation Scale [15] to assess the extent to which trainees embraced a uni-disciplinary (e.g., propensity to work within a single discipline), multi-disciplinary (e.g., working with other disciplines in a sequential or additive fashion), or interdisciplinary (e.g., working jointly with other disciplines to address a shared problem) orientation to their research. Additionally, students were asked to submit a brief online discussion reflecting on the process and outcomes of the collaborative team science session.

The Kolmogorov–Smirnov test showed that all variables were normally distributed, and paired sample *t*-tests were used for analyses. Although the sample size for this pilot study is comparatively small, the *t*-test is an appropriate statistic for normally distributed small and extremely small sample sizes [16].

## Initial Evidence of Impact on Learning

The sample (*n* = 20) consisted of 5 male and 15 female trainees. Twelve of the trainees reported their race as White, four as Asian, two as Black, and one as mixed; one of the participants reported Hispanic/Latino(a) ethnicity. Trainee majors included social behavioral (*n* = 7) and biomedical (*n* = 13) disciplines. The age of the trainees ranged from 22 to 35 (*M* = 27.25, *SD* = 4.02).

A paired *t*-test compared pre- and post-module questionnaire CRAI scores to test for improvement in self-assessment of research efficacy. Results indicated that participating in a collaborative skill-building workshop led to a statistically significant improvement in self-reported research efficacy (*t* = 10.59, *P* = 0.004). The level of research self-efficacy is likely to fluctuate as trainees progress through their careers and get exposed to new knowledge and technologies, but we argue that repeated exposure to such interventions would create long-lasting effects over time. Guided practice of collaboration and sustained increases in research self-efficacy can be maintained and incorporated into what trainees see as their core academic strengths and set of competencies. Similarly, a paired *t*-test comparing pre- and post-module scores showed a statistically significant increase of interdisciplinary research orientation (*t* = 5.56, *P* = 0.03) while showing no significant changes in uni- and multi-disciplinary research orientation scores.

The review of the discussion posts showed that students found it beneficial to articulate the contributions of their individual research programs. Throughout the process, they felt validation of their knowledge, skills, and research contribution. They also appreciated getting better understanding of how their programs of research intersected with questions pursued by students in other scientific disciplines, and how diverse scientific contributions fit within the larger societal needs to find solutions for pressing health problems. Students also commented that the session provided them with an opportunity to gain confidence in engaging in cross-disciplinary collaborations.

In conclusion, the early results of this workshop show that guided dialogues around *Strategic Team Science* increase research self-efficacy and interdisciplinary research orientation. Scaffolded collaboration dialogues allow students from diverse disciplines to engage actively and share ideas equitably. Furthermore, the approach described in this paper can serve as a tool for inter-generational knowledge and cross-disciplinary mentoring and knowledge sharing among established and emerging scholars. Future and larger-scale efforts could evaluate the extent to which guided practice and replicable models of collaborative communication build the capacity for innovative translational research and support the development of inclusive research practices through team science, mentoring, and coaching.
